# Integrated nomograms to predict overall survival and recurrence-free survival in patients with combined hepatocellular cholangiocarcinoma (cHCC) after liver resection

**DOI:** 10.18632/aging.103577

**Published:** 2020-08-13

**Authors:** Tao Wang, Xianwei Yang, Huairong Tang, Junjie Kong, Shu Shen, Haizhou Qiu, Wentao Wang

**Affiliations:** 1 Department of Liver Surgery and Liver Transplantation Center, West China Hospital of Sichuan University, Chengdu 610041, P. R. China; 2 Physical Examination Center, West China Hospital of Sichuan University, Chengdu 610041, P. R. China

**Keywords:** combined hepatocellular cholangiocarcinoma (cHCC), prognosis, least absolute shrinkage and selection operator (LASSO), satellite nodules (SAT), microvascular invasion (MVI)

## Abstract

The current clinical classification of primary liver cancer is unable to efficiently predict the prognosis of combined hepatocellular cholangiocarcinoma (cHCC). Accurate satellite nodules (SAT) and microvascular invasion (MVI) prediction in cHCC patients is very important for treatment decision making and prognostic evaluation. The aim of this work was to explore important factors affecting the prognosis of cHCC patients after liver resection and to develop preoperative nomograms to predict SAT and MVI in cHCC patients. The nomogram was developed using the data from 148 patients who underwent liver resection for cHCC patients at our hospital between January 2006 and December 2014. Based on the results of the multivariate analysis, a nomogram integrating all significant independent factors affecting overall survival and recurrence-free survival was constructed to predict the prognosis of cHCC. Next, risk factors for SAT and MVI were evaluated with logistic regression. Blood signatures were established using the LASSO regression, and then, we combined the clinical risk factors and blood signatures of the patients to establish predictive models for SAT and MVI. The C-index of the nomogram for predicting survival was 0.685 (95% CI, 0.638 to 0.732), which was significantly higher than the C-index for other liver cancer classification systems.

## BACKGROUND

Combined hepatocellular cholangiocarcinoma (cHCC) is a rare malignant liver tumor containing elements and clinicopathological features of both hepatocellular carcinoma (HCC) and intrahepatic cholangiocarcinoma (ICC). HCC-CCA tumors, which are thought to arise from hepatic progenitor cells (HPCs), occur in the presence of damaged hepatocytes and/or cholangiocytes. cHCC was first described by Allen and Lisa in 1949. Because there is no standardized classification system, the incidence of cHCC reported in the literature varies widely, accounting for 0.4% to 14.2% of all primary liver cancers [1–6]. In 2010, the World Health Organization (WHO) issued the latest definition and classification of cHCC, which became a widely accepted classification system used by hepatobiliary surgeons [7]. cHCC is divided into a classical type and subtypes with stem cell features. Moreover, the latter is subdivided into a typical subtype, intermediate cell subtype, and cholangiolocellular subtype. Some researchers believe that the biological behavior of cHCC is intermediate between the behavior of HCC and ICC, so its clinical prognosis is significantly worse than that of HCC, but better than that of ICC, [8] however, some scholars believe that the clinical prognosis of cHCC is significantly worse than that of ICC and HCC [5, 9]. Due to the rarity of cHCC and the essential characteristics that make it difficult to confirm a definitive diagnosis before surgery or biopsy, surgical resection is the most common treatment approach. According to previous studies, there is little information on the surgical outcomes and prognostic factors associated with malignancy. In addition, unlike HCC or ICC for which many preoperative prognostic prediction systems have been established, so far, there is still no effective prognostic model for this distinct hepatobiliary malignancy. It is not clear whether the current liver cancer classification system can predict the prognosis of patients with cHCC. Therefore, we retrospectively performed a comprehensive analysis of the clinicopathological characteristics and prognostic factors related to overall survival (OS) and recurrence-free survival (RFS) in cHCC patients in our single center. Moreover, we sought to develop and validate a novel nomogram that incorporates laboratory blood indicators for the preoperative prediction of important factors (including satellite nodules and microvascular invasion) that affect both RFS and OS in cHCC patients.

## RESULTS

### Clinical characteristics of the study patients

After careful reviews of the medical records, a total of 212 patients with cHCC confirmed by pathology who underwent curative liver resection were eligible for this study. All cHCC patients were followed up after initial treatment until December 2018. Among the entire set, the median OS was 16.5 months (range: 4.9–84.6 months). The 1-, 2- and 4-year OS rate were 79.7%, 27.4% and 8.5%, respectively. The demographic and clinical characteristics of the patients in the training and validation sets are listed in Table 1. The baseline demographic and clinical characteristics of the patients in the training and validation sets were similar (*P>*0.05). There was no significant difference in the cHCC patients RFS and OS between two groups (Supplementary Figure 1).

**Table 1:** Perioperative data.

	**Training set (n=148)**	**Validation set (n=64)**	***P* value**
**Age (years), median (range)**	52 (21-73)	52.5(36-78)	0.393
**Gender, (male/ female)**	125/23	49/15	0.169
**BMI (kg/m^2^), median (range)**	23.28(17.63-33.52)	22.22(18.18-29.09)	0.180
Metabolic syndrome*, n (%)			0.362
Yes	33(22.3%)	18(28.1%)	
No	115(77.7%)	46(71.9%)	
**Portal hypertension, n (%)**			0.403
Yes	38(25.7%)	20(31.25%)	
No	110(74.3%)	44(68.75%)	
**HbsAg positive, n (%)**			0.981
Yes	102(68.9%)	44(68.75%)	
No	46(31.1%)	20(31.25%)	
**HBV DNA positive, n (%)**			0.246
Yes	35(23.6%)	20(31.25%)	
No	113(76.4%)	44(68.75%)	
**Anti-HCV, positive, n (%)**			0.384
Yes	2(1.4%)	2(3.1%)	
No	146(98.6%)	62(96.9%)	
**Baseline laboratory investigations**			
WBC count, median (range) ×10^9^/L	5.77 (2.47-22.76)	5.98 (2.67-17.71)	0.446
NEUT count, median (range) ×10^9^/L	3.67 (1.37-18.19)	3.53(0.91-16.79)	0.611
PLT count, median (range), ×10^9^/L	141(23-399)	140 (31-458)	0.591
ALT (U/L), median (range)	37 (10-268)	32(11-272)	0.343
AST (U/L), median (range)	35(19-432)	36(13-567)	0.858
ALP (U/L), median (range)	108(45-718)	98(42-356)	0.392
GGT (U/L), median (range)	74(18-973)	67.5(18-282)	0.686
TBIL (umol/L), median (range)	13.3 (4.5-34.7)	12.4 (4.4-54.5)	0.209
PT(s), median (range)	12 (10.1-32.9)	11.95(9.8-24.3)	0.777
AFP level ng/mL	55.4 (0.96-47843)	33.4(1.6-18825)	0.748
CA19-9 level, U/mL	29.5(0.6-1000)	27.6(0.6-1000)	0.971
CEA level, ng/mL	2.43 (0.2-134.5)	2.37(0.61-41.88)	0.908
**ICG-R15(%)**	6.2(0.8-20.6)	5.4(0.9-22.7)	0.466
**Tumor size (cm), median (range)**	5.4(0.7-17.5)	4.9(1.5-13)	0.546
**Tumor number, (Multiple/solitary)**			0.651
multiple	35(23.6%)	17(26.6%)	
solitary	113(76.4%)	47(73.4%)	
**Tumor location**			0.656
Left lobe	42(28.4%)	16 (25%)	
Right lobe	84(56.7%)	38(59.4%)	
Both lobes	22(14.9%)	10(15.6%)	
**Extent of liver resection, n, (%) (major/ minor)**			0.631
major	92(62.2%)	42(65.6)	
minor	56(37.8%)	22(34.4%)	
**MVI, n, (yes/no)**			0.866
Yes	48 (32.4%)	20 (31.25%)	
No	100 (67.6%)	44 (68.75%)	
**Macroscopic vascular invasion, (yes/no)**			0.108
Yes	46 (31.1%)	13(20.3%)	
No	102 (68.9%)	51(79.7%)	
**Satellite nodules, n, (%)**			0.119
Yes	32 (21.6%)	8(12.5%)	
No	116(78.4%)	56(87.5%)	
**Lymph node metastasis, n, (%)**			0.786
Present	21(14.2%)	10(15.6%)	
Absent	127(85.8%)	54(84.4%)	
**Tumor encapsulation, n, (%)**			0.879
incomplete	70 (47.3%)	31(48.4%)	
complete	78 (52.7%)	33(51.6%)	
**Differentiation grade, n, (%)**			0.763
low	40(27%)	15(23.4%)	
Moderate/high	29(19.6%)	15(23.4%)	
Not evaluable	79(53.4%)	34(53.2%)	
**Ishak fibrosis score**			0.835
F1	74(50%)	31(48.4%)	
F0	74(50%)	33(51.6%)	
**Operation approach**			0.007
LLR	2(1.4%)	11(17.2%)	
OLR	134(90.5%)	48(75%)	
OLR+ RFA	12(8.1%)	5(7.8%)	
**Adjuvant chemotherapy**			0.226
Yes	34(23.0%)	10(15.6%)	
No	114(77%)	54(84.4%)	

Abbreviations: HBsAg, hepatitis B surface antigen; HBV, hepatitis B virus; HCV-Ab, Hepatitis C antibody; BMI, body mass index; WBC, white blood cell; NEUT, neutrophil; PLT, platelets; ALT, alanine aminotransferase; AST, aspartate transaminase; ALP, alkaline phosphatase; GGT, γ-glutamyl transferase; TBIL, Total bilirubin; PT, Prothrombin time; AFP, α- fetoprotein; CA19-9, carbohydrate antigen 19-9; CEA, carcinoembryonic antigen; ICG-R15, indocyanine green retention rate at 15 minutes; LLR, laparoscopic liver resection; OLR, open liver resection; RFA, radiofrequency ablation.

### Independent factors significantly associated with OS and RFS

**Figure 1:**
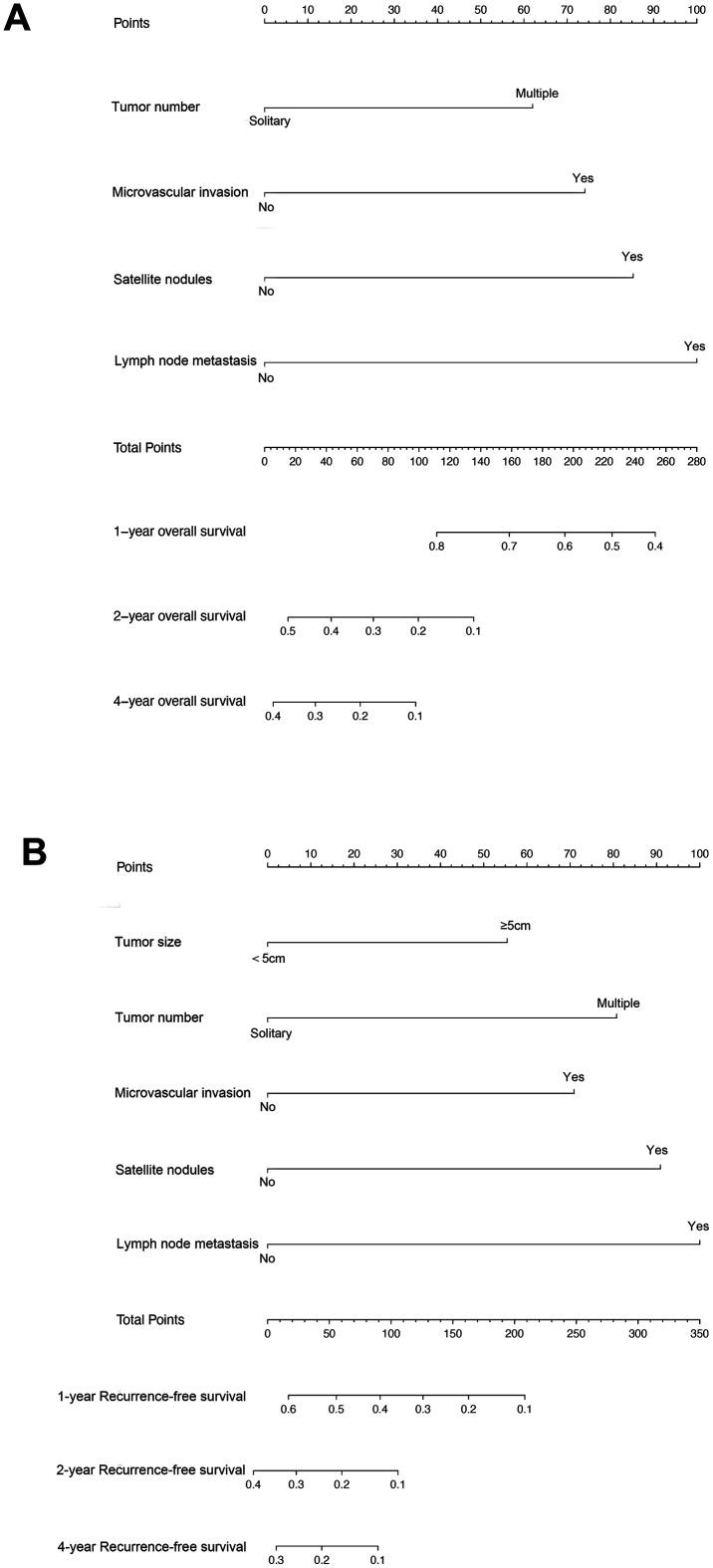
Combined hepatocellular cholangiocarcinoma (cHCC) overall survival (**A**) and recurrence-free survival (**B**) nomogram. The sum of these numbers is located on the total points axis, and a line is drawn upward to determine the number of points received for each variable value. A line is drawn downward to the survival axes to determine the likelihood of 1-, 2- or 4-year survival).

The results of univariate and multivariate Cox regression analyses of RFS and OS after curative resection of cHCC are listed in Table 2 and Table 3. In the univariate analysis of OS in the training set, sex, maximum tumor size, multiple tumors, MVI, macroscopic vascular invasion, SAT, LN metastasis were associated with shorter OS (all *P* < 0.05, Table 2), Parameters with *P* < 0.05 in univariate analyses were subjected to the multivariate Cox regression model for stepwise variable selection to determine the prognostic factors. Multivariate Cox regression analysis identified that tumor number (multiple vs. single, HR,1.851; 95% CI, 1.188-2.883; *P* =0.006), MVI (Yes vs. No, HR,1.938; 95% CI, 1.273-2.950; *P*=0.002), SAT (Yes vs. No, HR, 2.152; 95% CI,1.336-3.466; *P*=0.002), and LN metastasis (Yes vs. No, HR, 2.397; 95% CI,1.391-4.131; *P*=0.002) were independent predictors of OS in patients after curative resection of cHCC, as listed in Table 2. The univariate analysis of RFS in the training set showed that age, maximum tumor size, tumor number, MVI, macroscopic vascular invasion, SAT, LN metastasis were associated with an increased tumor recurrence rate (all *P*< 0.05). Multivariate Cox regression analysis identified that maximum tumor size (>5 vs. ≤5 cm, HR,1.507; 95% CI, 1.030-2.204; *P*=0.035), tumor number (multiple vs. single, HR,1.972; 95% CI, 1.290-3.016; *P* =0.002), MVI (Yes vs. No, HR, 1.528; 95% CI,1.024-2.279; *P*=0.038), SAT (Yes vs. No, HR, 1.953; 95% CI,1.259-3.029; *P*=0.003), and LN metastasis (Yes vs. No, HR, 2.059; 95% CI,1.228-3.453; *P*=0.006) were independent predictors of RFS in patients after curative resection of cHCC, as listed in Table 3. Based on the results of the multivariate analysis, a nomogram integrating all significant independent factors was constructed to predict OS and RFS for cHCC patients, as shown in Figure 1. The C-index of prediction of OS and RFS in training set were 0.685 (95% CI, 0.638 to 0.732) and 0.685 (95% CI, 0.639 to 0.731), respectively. The C-index of prediction of OS and RFS in validation set were 0.654 (95% CI, 0.567 to 0.741) and 0.669 (95% CI, 0.582 to 0.756), respectively.

**Table 2:** Univariate and multivariate analysis on the overall survival of all patients (N = 148) in the training set.

**Overall survival Variable**	**Univariate analysis**				**Multivariate analysis**		
	**Hazard ratio**	**95% CI**	***P* value**		**Hazard ratio**	**95% CI**	***P* value**
Age (≥55 vs. <55 years)	0.849	0.578-1.248	0.405		0.962	0.647-1.431	0.850
Sex (female vs. male)	**0.463**	**0.247-0.867**	**0.016**		0.529	0.280-1.001	0.050
Metabolic syndrome^*^ (yes vs. no)	0.914	0.575-1.452	0.704				
HBsAg (positive vs. negative)	1.399	0.906-2.161	0.130				
HBV DNA load (positive vs. negative)	1.122	0.710-1.774	0.621				
Portal hypertension (present vs. absent)	1.206	0.806-1.806	0.362				
AFP (>55 vs. ≤55 ng/mL)	0.965	0.657-1.416	0.855				
CA19-9 (>29.5 vs. ≤29.5 U/mL)	1.277	0.869-1.876	0.213				
CEA (>2.4vs. ≤2.4ng/mL)	1.116	0.760-1.638	0.575				
Maximum tumor size (>5 vs. ≤5cm)	**1.716**	**1.149-2.562**	**0.008**		1.424	0.934-2.173	0.101
Tumor number (multiple vs. single)	**2.008**	**1.303-3.094**	**0.002**		**1.851**	**1.188-2.883**	**0.006**
Differentiation grade (low vs. moderate)	1.176	0.711-1.947	0.528				
Tumor encapsulation (incomplete vs. complete)	1.313	0.894-1.929	0.165				
MVI (yes vs. no)	**2.110**	**1.420-3.134**	**<0.001**		**1.938**	**1.273-2.950**	**0.002**
Macroscopic vascular invasion (yes vs. no)	**1.658**	**1.109-2.479**	**0.014**		1.244	0.810-1.908	0.318
Satellite nodules (yes vs. no)	**2.750**	**1.748-4.326**	**<0.001**		**2.152**	**1.336-3.466**	**0.002**
Lymph node metastasis (yes vs. no)	**2.901**	**1.709-4.925**	**<0.001**		**2.397**	**1.391-4.131**	**0.002**
Ishak fibrosis score (F1 vs. F0)	1.250	0.850-1.838	0.257				
Extent of liver resection (major vs. minor)	1.151	0.770-1.720	0.494				
Adjuvant chemotherapy (yes vs. no)	0.879	0.564-1.370	0.570				

Abbreviations: CI: confidence interval; HBsAg, hepatitis B surface antigen; HBV, hepatitis B virus; AFP, α- fetoprotein; CA19-9, carbohydrate antigen 19-9; CEA, carcinoembryonic antigen; MVI, microvascular invasion; * the patients had at least one of following disease: diabetes mellitus, hypertension, hyperlipidemia; Bold indicates statistically significant difference.

**Table 3:** Univariate and multivariate analysis on the recurrence-free survival of all patients (n=148) in the training set.

**Overall survival Variable**	**Univariate analysis**				**Multivariate analysis**		
	**Hazard ratio**	**95% CI**	***P* value**		**Hazard ratio**	**95% CI**	***P* value**
**Age (≥52 vs. <52 years)**	**0.672**	**0.470-0.960**	**0.029**		0.763	0.528-1.102	0.149
Sex (female vs. male)	0.687	0.417-1.135	0.143		0.836	0.501-1.395	0.493
Metabolic syndrome^*^ (yes vs. no)	0.875	0.571-1.341	0.540				
HBsAg (positive vs. negative)	1.387	0.940-2.047	0.099				
HBV DNA load (positive vs. negative)	1.137	0.749-1.725	0.547				
Portal hypertension (yes vs. no)	1.168	0.801-1.704	0.419				
AFP (>55 vs. ≤55 U/mL)	0.975	0.685-1.388	0.890				
CA19-9 (>29.5 vs. ≤29.5 U/mL)	1.093	0.768-1.555	0.622				
CEA (>2.4vs. ≤2.4ng/mL)	0.956	0.672-1.361	0.804				
Maximum tumor size (>5 vs. ≤5cm)	**1.808**	**1.249-2.618**	**0.002**		**1.507**	**1.030-2.204**	**0.035**
Tumor number (multiple vs. single)	**1.938**	**1.287-2.917**	**0.002**		**1.972**	**1.290-3.016**	**0.002**
Differentiation grade (low vs. moderate)	1.176	0.711-1.974	0.528				
Hepatic capsule (incomplete vs. complete)	1.238	0.869-1.765	0.237				
MVI (yes vs. no)	**1.874**	**1.287-2.729**	**0.001**		**1.528**	**1.024-2.279**	**0.038**
Macroscopic vascular invasion (yes vs. no)	**1.742**	**1.197-2.535**	**0.004**		**1.397**	**0.937-2.083**	**0.101**
Satellite nodules (yes vs. no)	**2.463**	**1.609-3.769**	**<0.001**		**1.953**	**1.259-3.029**	**0.003**
Lymph node metastasis (yes vs. no)	**2.376**	**1.438-3.925**	**0.001**		**2.059**	**1.228-3.453**	**0.006**
Ishak fibrosis score	1.241	0.872-1.768	0.231				
Extent of liver resection (major vs. minor)	1.134	0.787-1.634	0.501				
Adjuvant chemotherapy (yes vs. no)	1.002	0.669-1.500	0.992				

Abbreviations: CI: confidence interval; HBsAg, hepatitis B surface antigen; HBV, hepatitis B virus; AFP, α- fetoprotein; CA19-9, carbohydrate antigen 19-9; CEA, carcinoembryonic antigen; MVI, microvascular invasion;

* the patients had at least one of following disease: diabetes mellitus, hypertension, hyperlipidemia;

Bold indicates statistically significant difference.

As shown in Supplementary Figure 2, the BCLC stage, the 8^th^ edition HCC AJCC TNM stage, the 8^th^ edition ICC AJCC TNM stage, and CNLC stage have had good prognostic stratification ability for patients between stage I and later stages in the training group. However, the current stage systems did not perform well in the prognostic stratification of advanced cHCC with later stages in the training group.

The C-index of the nomogram predicting OS in the training set was significantly higher than that of the BCLC staging system(0.601, 95% CI: 0.547 to 0.655, *P<*0.001), 8^th^ edition AJCC HCC TNM staging system(0.625, 95% CI: 0.574 to 0.676, *P*= 0.013), 8^th^ edition AJCC ICC TNM staging system(0.593, 95% CI: 0.542 to 0.644, *P<*0.001), and CNLC staging system(0.604, 95% CI: 0.550 to 0.658, *P*<0.001). The calibration plot for the probability of OS and RFS at 1, 2 or 4-years after surgery showed an optimal agreement between the nomogram prediction and actual observation in the training set and validation set, as shown in Supplementary Figure 3. Furthermore, we found that our nomogram had a better net benefit across a wider scale of threshold probabilities for predicting 1-, 2- and 4-year overall survival than the BCLC staging system, the 8^th^ edition AJCC staging system (HCC and ICC), CNLC staging system in the DCA, as shown in Supplementary Figure 4.

### Independent factors significantly associated with the presence of SAT and MVI

**Figure 2:**
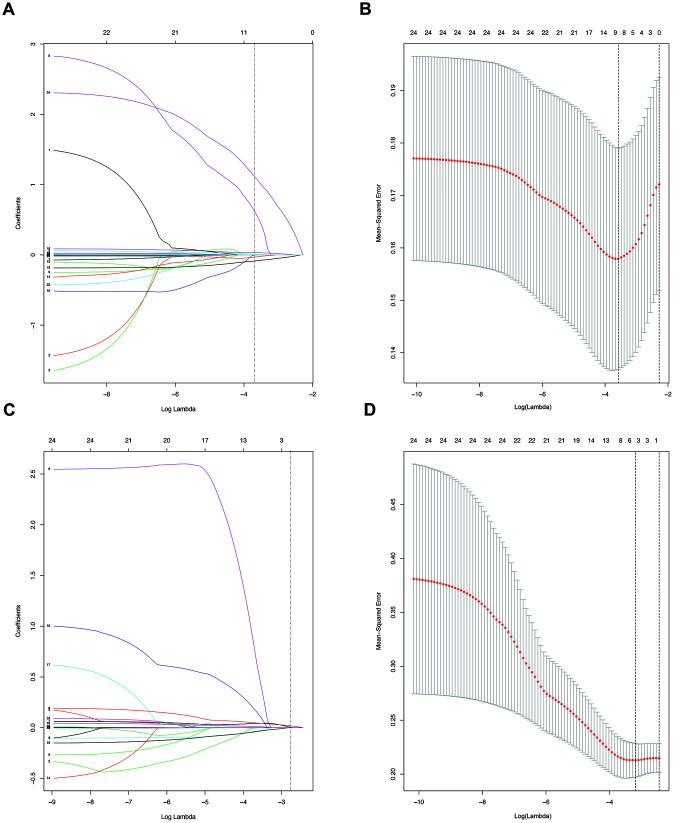
(**A**). LASSO coefficient profiles of the nine selected blood signatures for SAT. A dashed vertical line is drawn at the value (logγ=-3.6) chosen by 10-fold cross-validation. (**B**). Partial likelihood deviance for the LASSO coefficient profiles. A light dashed vertical line stands for the minimum partial likelihood deviance. A dashed vertical line stands for the partial likelihood deviance at the value (logγ=-3.6). (**C**). LASSO coefficient profiles of the three selected blood signatures for MVI. A dashed vertical line is drawn at the value (logγ=-2.8) chosen by 10-fold cross-validation. (**D**). Partial likelihood deviance for the LASSO coefficient profiles. A light dashed vertical line stands for the minimum partial likelihood deviance. A dashed vertical line stands for the partial likelihood deviance at the value (logγ=-2.8).

**Figure 3:**
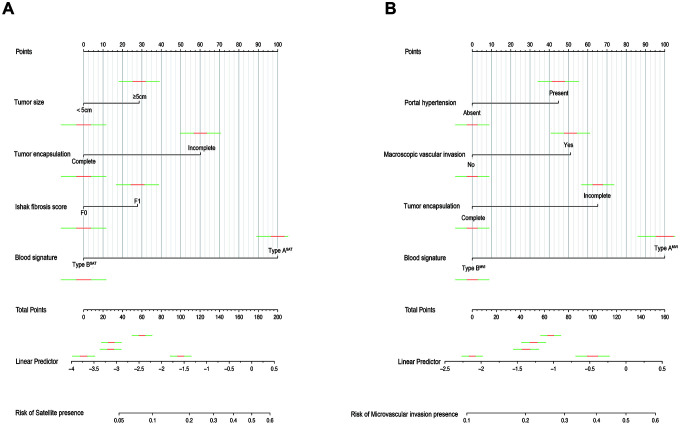
**Nomograms for predicting SAT and MVI and the calibration plot.** (**A**). The nomogram maps the predicted probability of SAT on a scale of 0 to 200. (**B**). The nomogram maps the predicted probability of MVI on a scale of 0 to 160. For each covariate, a vertical line is drawn upwards and the corresponding points are noted. This is repeated for each covariate, ending with a total score that corresponds to a predicted probability of SAT or MVI at the bottom of the nomogram.

**Figure 4:**
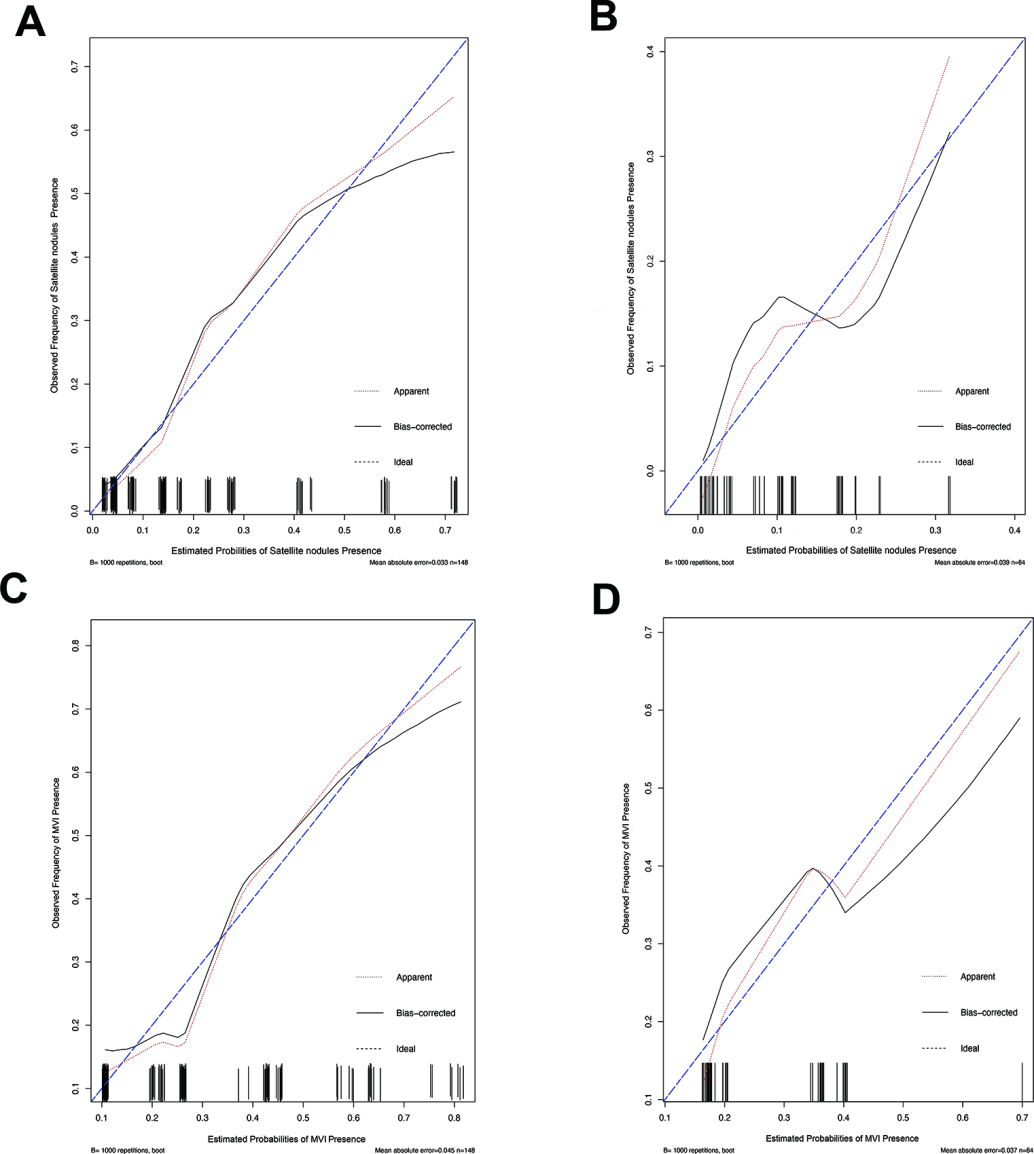
**The calibration curve for predicting patient MVI and SAT in the training set and in the validation set.** The C-index value for the nomogram predicting satellite nodules was 0.826 (95% CI, 0.743 to 0.909) for the training set (**A**) and 0.778(95% CI, 0.630 to 0.926) for the validation set (**B**), while the C-index value for the nomogram predicting MVI was 0.771 (95% CI, 0.688 to 0.854) for the training set (**C**) and 0.702 (95% CI, 0.572 to 0.832) for the validation set (**D**). Ideal line (blue), estimated probabilities correspond to the actual observation; apparent line (red), predictive capability of the model obtained after data analysis; bias-corrected line, predictive capability of the model obtained after bootstrap correction.

**Figure 5:**
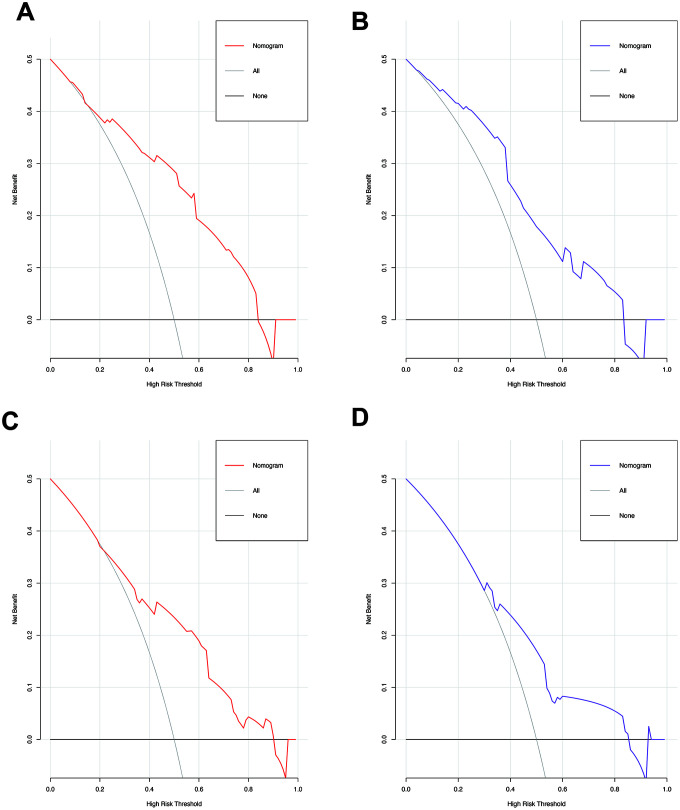
The decision curves of the nomograms for predicting the presence of SAT and MVI in the training (**A**, **C**) and validation sets (**B**, **D**). The Y-axis represents the net benefit. The X-axis shows the threshold probability. The horizontal solid black line represents the hypothesis that no patients experienced presence of SAT or MVI, and the solid gray line represents the hypothesis that all patients met the endpoint.

Furthermore, in order to predict SAT and MVI well before surgery and guide clinical decision making, univariate logistic regression analysis was performed to estimate the impacts of clinical and imaging features on the presence of SAT and MVI in the training set. According to univariable logistic regression analysis, maximum tumor size, tumor encapsulation, and the Ishak fibrosis score were associated with SAT. Additionally, univariate logistic regression analysis showed that portal hypertension, MCI, tumor encapsulation, and the Ishak fibrosis score were associated with MVI. Stepwise multivariate logistic regression analysis was further performed to identify significant independent risk factors. The multivariate analyses revealed that maximum tumor size (>5 vs. ≤5 cm, OR, 2.484, 95%CI, 1.011-6.107, *P* =0.047), tumor encapsulation (OR, 2.914, 95%CI, 1.239-5.725, *P* =0.014), Ishak fibrosis score (OR, 2.421, 95% CI, 1.024-5.725, *P*=0.044) were identified as independent risk factors for (Table 4). In addition, portal hypertension (OR, 2.477, 95%CI, 1.073-5.718, *P* =0.034), MCI(OR, 2.406, 95%CI, 1.067-5.423, *P* =0.034), hepatic encapsulation (OR, 2.563, 95%CI, 1.171-5.611, *P* =0.019) were independently associated with MVI (Table 5).. To elucidate and synthetically estimate the significant values for the blood indexes, the LASSO logistic regression algorithm was used to select the candidate blood index, Using the coefficients derived from the LASSO logistic regression models in the training set, we then constructed a formula to calculate for each patient. The LASSO coefficient profiles of the selected blood features are shown in Figure 2. The blood signature score is based on their personalized levels of the 25 blood features, where the blood-satellite score=0.007× activated partial thromboplastin time (APTT) -0.001× platelet (PLT) +0.523×international normalized ratio (INR)-0.257×total bilirubin (TBIL)-0.003×aspartate transaminase(AST)+0.031×globulin (GLB)-0.081 × albumin (ALB) + 0.001 × CA199 + 1.053 × HBV-DNA +0.427, and the blood-MVI score = 0.037×prothrombin time (PT)+0.032×TBIL-0.016×ALB-1.105, as listed in Supplement Table 1. Using the ROC curve, we classified patients into a type A^SAT^ group and type B^SAT^ group with a blood signature score of -1.228 as the cut-off value. We further classified patients into a type A^MVI^ group and type B^MVI^ group with a blood signature score of -0.605 as the cut-off value, as shown in Supplementary Figure 5. Based on the results of the blood signatures and multivariate logistic regression, nomogram for predicting SAT and MVI was established (Figure 3). The model had good predictive ability for SAT and MVI. The C-index for the nomogram for the prediction of SAT was 0.826 (95% CI, 0.743 to 0.909) for the training set and 0.778 (95% CI, 0.630 to 0.926) for the validation set, while the C-index for the nomogram for the prediction of MVI was 0.771 (95% CI, 0.688 to 0.854) for the training set and 0.702 (95% CI, 0.572 to 0.832) for the validation set. The calibration curve for nomograms showed good agreement between predictions and observations for SAT prediction in the training set and validation set, as well as for MVI prediction in the training set and validation set (Figure 4). The decision curves of the nomograms for predicting the presence of SAT and MVI are presented for the training set and validation set. (Figure 5).

**Table 4:** Logistic regression models of variables associated with SAT.

**Variable**	**Univariate regression model**				**Multivariate regression model**		
	**Odds ratio**	**95% CI**	***P* value**		**Odds ratio**	**95% CI**	***P* value**
Age (≥55 vs. <55 years)	0.541	0.242-1.208	0.134				
Sex (female vs. male)	0.497	0.138-1.791	0.285				
Metabolic syndrome^*^ (yes vs. no)	0.969	0.377-2.493	0.948				
Portal hypertension (yes vs. no)	1.164	0.508-2.666	0.719				
Maximum tumor size (≥5 vs. <5cm)	**2.150**	**0.916-5.044**	**0.079**		**2.484**	**1.011-6.107**	**0.047**
Tumor number (multiple vs. single)	2.088	0.852-4.730	0.111				
Macroscopic vascular invasion (yes vs. no)	1.389	0.613-3.147	0.432				
Tumor encapsulation (incomplete vs. complete)	**3.117**	**1.354-7.175**	**0.008**		**2.914**	**1.239-5.725**	**0.014**
Ishak fibrosis score (F1 vs. F0)	**2.269**	**1.004-5.131**	**0.049**		**2.421**	**1.024-5.725**	**0.044**

Abbreviations: SAT, satellite nodules; CI: confidence interval; Bold indicates statistically significant difference

* the patients had at least one of following disease: diabetes mellitus, hypertension, hyperlipidemia;

**Table 5:** Logistic regression models of variables associated with MVI.

**Variable**	**Univariate regression model**				**Multivariate regression model**		
	**Odds ratio**	**95% CI**	***P* value**		**Odds ratio**	**95% CI**	***P* value**
Age (≥55 vs. <55 years)	**0.491**	**0.243-0.993**	**0.048**		0.523	0.236-1.158	0.110
Sex (female vs. male)	0.388	0.124-1.211	0.103				
Metabolic syndrome^*^ (yes vs. no)	**0.386**	**0.148-1.011**	**0.053**		0.442	0.160-1.222	0.116
Portal hypertension (yes vs. no)	**2.913**	**1.406-6.039**	**0.004**		**2.477**	**1.073-5.718**	**0.034**
Maximum tumor size (≥5 vs. <5cm)	1.704	0.831-3.491	0.146				
Tumor number (multiple vs. single)	1.317	0.596-2.911	0.496				
Macroscopic vascular invasion (yes vs. no)	**2.679**	**1.291-5.560**	**0.008**		**2.406**	**1.067-5.423**	**0.034**
Tumor encapsulation (incomplete vs. complete)	**3.263**	**1.583-6.726**	**0.001**		**2.563**	**1.171-5.611**	**0.019**
Ishak fibrosis score (F1 vs. F0)	**2.417**	**1.186-4.925**	**0.015**		1.565	0.701-3.496	0.275

Abbreviations: MVI: microvascular invasion; CI: confidence interval; Bold indicates statistically significant difference.

* the patients had at least one of following disease: diabetes mellitus, hypertension, hyperlipidemia;

## DISCUSSION

cHCC is a rare distinct type of primary liver cancer (PLC), it is not simply as a combination of ordinary HCC and ICC but rather is composed of phenotypical components of both HCC and cholangiocarcinoma (CC) [9–12]. The typical pathological manifestations of cHCC are dual hepatocellular and biliary differentiation with the two types of tumor cells intermingling and transition zones with intermediate cellular morphology, with distinct immunohistochemical features demonstrating malignant transformation in both hepatic and biliary cells [11–13]. The histogenesis and natural history of cHCC remain unclear. It is increasingly believed that cHCC may originate from HPCs, which are intermediate stem cells capable of undergoing bidirectional differentiation into hepatocytes and bile duct epithelial cells [14, 15]. Coulouarn et al determined that the occurrence of cHCC might be related to the microenvironment remodeling, and the TGFβ and Wnt/β-catenin pathways were identified as the two major activated signaling pathways in cHCC [15]. The TGFβ pathway is related to biliary differentiation and in epithelial-mesenchymal transition (EMT); the Wnt/β-catenin pathway plays an important role in preventing them from differentiating into the hepatocyte lineage and guiding them to differentiate into biliary duct cells during liver embryonic growth [16, 17]. In addition, a recent study showed that mutations in genes *KRAS, ARID1A, TERT promoter, TP53*, and *CTNNB1* might also be associated with cHCC using the targeted gene panel with genomic and transcriptomic profiling [18, 19]. Further studies are needed to investigate the pathogenesis of cHCC.

Some researchers have suggested that the biological features of cHCC resemble those of HCC, however, other investigators reported that the clinical features of cHCC were more similar to those of ICC [20–22]. Therefore, they have previously classified cHCC as HCC or ICC to explore its prognosis. There is no effective specialized predictive staging system for cHCC, and the existing predictive models for HCC and ICC did not have good predictive ability for cHCC. Moreover, none of these systems were specifically developed for postoperative prognostic prediction. We observed that the C-index of these systems varied from 0.593 to 0.625 for the prediction of OS in the training cohort. The predictive accuracy of these systems for patients with cHCC who undergo curative liver resection might be affected by these issues.

Complete surgical liver resection is still the major curative treatment for cHCC [8, 23–27]. Some studies have demonstrated that cHCC tends to have more aggressive behavior and a worse prognosis in comparison with HCC and ICC. However, these studies have been limited to case reports or case series. Due to the rarity of these malignancies, clinical prognostic medical data especially regarding important factors that affecting prognosis after radical surgical resection on the prognosis, are very limited. To our knowledge, our study is the most comprehensive comparison reported to date about the clinical characteristics and prognosis of cHCC patients after surgery, focusing on the recurrence and survival after surgical resection. Furthermore, we developed and validated a predictive model that incorporates the clinical risk factors and laboratory blood indicators for the preoperative prediction of SAT and MVI which are important factors affecting both OS and RFS.

We observed that the median OS following surgical resection for cHCC patients was 16.5months, and the 1-, 2-, and 4-year survival rates were 79.7%, 27.4%, and 8.5%, respectively. We found that most cHCC patients were male, and were likely to be older than 50 years (59.9%) when they were diagnosed; the results were similar to previous findings [8, 23–27]. The prognostic predictors in cHCC patients undergoing curative liver resection have not been well established. In the present study, multivariate analysis showed that a larger maximum tumor size, multiple tumors, MVI, SAT, and LNM were independent predictors for poor survival. In the past, tumor size and tumor number were considered to be important prognostic factors of PLC and have been included in various surgical staging systems for PLC. This may be related to the fact that the larger tumors and multiple tumors usually grow faster, have aggressive biological behavior, are more likely to break through the encapsulation to infiltrate surrounding liver tissue and are more prone to intrahepatic metastases. Consistent with previous studies in HCC, MVI and SAT which cannot be obtained prior to the resection of the tumor, are histological features related to aggressive biological behavior and poor survival outcomes [28–31]. MVI was reported to be related to the secretion of cytokines and proteins that promote angiogenesis by stromal cells in the tumor microenvironment, and the macroscopic type of the main tumor affects the occurrence of SAT [31, 32]. When the MVI and SAT are present, the tumor is more likely to have intrahepatic metastasis and recurrence through the portal vein. If we can identify the status of MVI and SAT before surgery, we can perform a comprehensive assessment to choose a wider surgical margin, anatomic liver resection, or even liver transplantation depending on the patient's condition. With the development of radiological technology, the histologic status can be diagnosed before surgery [33, 34]. However, the diagnosis of cHCC and the differentiation of cHCC from other PLCs based on imaging findings can be challenging because of the histologic diversity and complexity of cHCC components and the overlap of imaging characteristics of HCC and ICC [35–37]. Therefore, it is difficult to identify some pathological factors that affect prognosis based on imaging. Chae et al demonstrated that the variable ^18^F-fluorodeoxyglucose (FDG) uptake and a high tumor-to-normal liver standardized uptake value ratio (TLR) in cHCC are closely related to the molecular features of aggressive biological behavior by 18F-fluorodeoxyglucose positron emission tomography/computed tomography (PET/CT), but this technique is expensive, limiting its clinical applicability [38]. Although previous studies have revealed some factors that are associated with the presence of MVI and SAT, there is no direct way to predict them in routine clinical practice. Given that MVI and SAT have significant impacts on recurrence and survival after liver resection, a preoperative means of assessing the probability of MVI and SAT is needed. Therefore, we aimed to develop a simple and straightforward method that could be used in daily clinical practice to accurately predict pathological information preoperatively, rather than being limited to the identification of clinicopathological risk factors in resected specimens. Two predictive nomograms were developed and validated herein to predict SAT and MVI in patients with cHCC. The nomogram for SAT includes four factors: tumor size, tumor encapsulation, Ishak fibrosis score obtained by biopsy, and blood signature obtained from LASSO regression. The nomogram for MVI incorporates four factors: portal hypertension, macroscopic vascular invasion, tumor encapsulation, and the blood signature. Both nomograms demonstrated good agreement between the predictions and observations in the training and validation sets.

In addition to MVI and SAT, the presence of LNM was another factor related to poor prognosis, and early extrahepatic recurrence was reported mainly in the lymph nodes of cHCC patients. To increase the R0 resection rate and improve the survival, we should perform lymph node dissection on patients suspected of having regional lymphadenopathy based on preoperative imaging findings. Transarterial chemoembolization (TACE) has been proven to increase the survival of HCC patients, but there is still controversy regarding the treatment effect of TACE on cHCC. In our study, TACE was found to have no effect on preventing tumor recurrence or prolonging OS. This may be related to the relatively fewer blood vessels and higher fibrosis in cHCC [39]. However, Seong et al found that the cHCC with global enhancement patterns on dynamic imaging showed a better response to TACE and prognosis [40].

A predictive nomogram for cHCC was constructed based on the results of multivariate analysis. The nomogram we established includes postoperative pathological factors, which have not been included in the other staging systems. The nomogram performed well with regard to predicting survival, and its predictive ability was assessed with the C-index (0.685 for the training and validation sets, respectively) and the calibration curve. When compared with the other HCC or ICC staging systems, the nomogram showed better predictive accuracy for survival.

Our study had several inherent limitations. First, the data for the training set and validation set came from a single center, which might have hampered the identification of possibly important predictive factors. The possibility of selection bias is another potential limitation of this study. Previous research reports are mainly limited to Asia, and we need to obtain data from multiple centers, especially European and American medical centers, to build external validation datasets and investigate the clinicopathological characteristics and prognostic factors of cHCC. Second, previous research has shown that increased tumor heterogeneity in cHCC might be considered a poor prognostic factor. However, in our study, we did not divide cHCC into various subtypes according to the latest classification of cHCC [11] and did not explore the impact of different pathological subtypes of cHCC on the postoperative prognosis because the morphological appearance and immunohistochemical characteristics of the stem cell components can be similar to the phenotypes of typical HCC and ICC. In particular, immunohistochemical markers might be not completely sensitive or specific to progenitor stem cells, and stem cell characteristic variants are very challenging to diagnose pathologically. Therefore, we need more clinical data specimens to explore the correlations between pathological subtypes and pathological risk factors and their impact on prognosis. Third, it is worth noting that the predictive nomogram we constructed was not satisfactory for the prediction of long-term survival. This may be related to the diversity of treatment after hepatectomy and the small sample size in our study. The subjects we included were patients undergoing curative liver resection, so whether our nomogram can be applied to patients who received treatment other than curative liver resection remains to be determined. In addition, whether advanced cHCC should be treated surgically still needs further exploration and research.

In conclusion, we have found important factors affecting prognosis after liver resection for cHCC, Furthermore, we constructed and validated a nomogram predicting the prognosis of cHCC. A nomogram was established that can objectively and accurately predict the preoperative risks of SAT and MVI based on clinical risk factors identified with LASSO regression.

## MATERIALS AND METHODS

### Patients and study design

**Figure 6:**
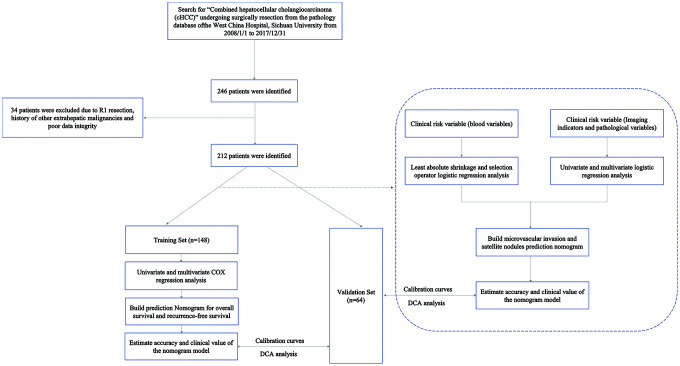
**The flowchart of patient selection.**

A total of 212 consecutive patients (174 men, 38 women) who underwent curative liver resection for cHCC between January 2006 and December 2017 at West China Hospital were enrolled in this study. The inclusion criteria were as follows: (1) liver resection, with tumor tissues pathologically confirmed as cHCC mixed cancer, not double cancer type or collision cancer; (2) Child-Pugh A or B7 (score ≤7 [less than or equal to]) liver function; (3) curative liver resection, defined as the complete removal of all macroscopic nodules with a clear margin (R0 resection); and (4) available detailed clinical characteristics. The exclusion criteria were (1) a history of extrahepatic malignancies and (2) poor clinical data integrity. Eligible patients(n=212) who underwent surgery were assigned to the training and validation sets at a ratio of 7:3 according to the scanning date: the early data before the 70 percent scanning date were allocated to the training set(n=148) for the development of the nomogram, whereas the other patients were allocated to the validation set(n=64) for the verification of the nomogram. The flowchart of this present study selection is shown in Figure 6 and the clinicopathologic characteristics of patients in the training and validation sets are listed in Table 1. This study obtained ethics approval from the ethics committee of Sichuan University and was performed in accordance with the 1975 Declaration of Helsinki. Written informed consent was obtained from each participant in the study.

### Data collection

The clinical medical data of cHCC patients who underwent curative liver resection were retrospectively collected from our hospital and included demographics, comorbid illnesses, portal hypertension, liver and renal function tests, hepatitis B and C immunology, HBV-DNA load, preoperative α-fetoprotein (AFP) level, preoperative serum carbohydrate antigen 19-9 (CA19-9) level, preoperative serum carcinoembryonic antigen (CEA) level, imaging data of tumors (including the maximum tumor size, tumor number, tumor location, and encapsulation status), pathological results of cHCC (including the differentiation grade, microvascular invasion (MVI), SAT, LN metastasis and Ishak fibrosis score), and surgery-related factors including the extent of liver resection (major or minor), intraoperative blood transfusion (yes or no), Barcelona Clinic Liver Cancer (BCLC) stage, 8^th^ American Joint Committee on Cancer (AJCC) TNM clinical stage(HCC and ICC) and China liver cancer (CNLC) stage [41–43]. Comorbid illnesses included diabetes mellitus, hypertension, and hyperlipidemia. Portal hypertension was defined by the presence of either esophageal varices or splenomegaly with a decreased platelet count (100 × 10^9^/L or less). MVI was defined as the presence of tumor in a portal vein, hepatic vein, or a large capsular vessel of the surrounding hepatic tissue lined by endothelium that was visible only on microscopy [44]. Macroscopic vascular invasion included major hepatic vessel invasion, defined as invasion of the first-and second-order branches of the portal veins or hepatic arteries, or as invasion of one or more of the three hepatic veins. Major resection was defined as resection of 3 or more Couinaud segments, while minor resection was defined as resection of fewer than 3 Couinaud segments [45]. The Ishak scoring system uses a 0-6 scale; F0 is defined as a fibrosis score 0-4 (no to moderate fibrosis), and F1 is defined as a fibrosis score 5 -6 (severe fibrosis or cirrhosis);

The extent of liver resection was determined according to the location of the tumor, tumor diameter, liver function and indocyanine green retention rate at 15 minutes (ICG-R15). The resection of the liver parenchyma was performed with an ultrasonic scalpel, CUSA, monopolar electrocoagulation, LigaSure, Endo-GIA and clips. Intermittent Pringle manipulation or a selective vascular clamp was used if necessary. Regional lymph nodes were dissected if metastasis was was suspected or diagnosed preoperatively or found intraoperatively. Intraoperative ultrasonography was undertaken routinely to identify lesion(s) and the relationship to surrounding vascular and biliary structures and determine whether there were still additional lesions that could not be seen by preoperative imaging findings in the remnant liver.

### Follow-up and recurrence treatment

In general, all patients who received curative liver resection were prospectively followed up through outpatient clinic visits or phone calls at intervals of 2-3 months during the first year after operation and 3-6 months thereafter. Chest CT examination and bone scintigraphy were performed when extrahepatic cHCC recurrence was suspected. Recurrent cHCC was treated with postoperative adjuvant therapy, repeated liver resection, radiofrequency ablation, and liver transplantation, depending on the status of the cHCC and liver function at the time of recurrence. In addition, for patients with high-risk factors for tumor recurrence, we recommended patients to undergo adjuvant therapy after surgery. Postoperative adjuvant therapy included systemic chemotherapy (mainly 5-fluorouracil) and transarterial chemoembolization (TACE). Survival information, including OS and RFS, was collected until December 31, 2018. OS was defined as the interval between resection and death, or the period up to the observation point. RFS was identified as the interval between resection and the recurrence of the primary tumors detected by dynamic radiological findings including intrahepatic recurrence and extrahepatic metastasis. The OS and RFS were measured in months.

### Risk factors for OS and RFS

Univariate and multivariate Cox regression analyses were used to identify the independent risk factors of cHCC. Parameters with *P* < 0.05 in univariate analyses were included in the multivariate Cox regression model for stepwise variable selection to determine the prognostic factors. A nomogram was constructed based on the results of multivariate analysis. A calibration curve was used to describe the consistency of the nomogram predictions of 1-year, 2-year, and 4-year OS and RFS with the actual values. Harrell's concordance index (C-index) was used to quantify the performance of this nomogram. Bootstraps with 1,000 resamples were used for these analyses. In addition, we used clinical data from the validation set for validation. The total points for each patient in the validation set were calculated with the established nomogram, and the C-index and calibration curve were derived based on the regression analysis. Decision curve analysis (DCA) was used to determine the clinical application value of the nomogram models by calculating the net benefits at each risk threshold.

### Risk factors for presence of satellite nodules and MVI

The least absolute shrinkage and selection operator (LASSO) logistic regression model was used to build a prognostic classifier for SAT and MVI in the training set, which integrated all types of serological variables. Using the coefficients derived from the LASSO logistic regression models, we then constructed a formula to calculate a score for each patient. Formula=expressionindex1× βindex1+…+ ex-pressionindexn × βindexn (where β is the regression coefficient derived from LASSO regression). We use the receiver operating characteristic (ROC) curve with calculations of the area under the curve (AUC) to determine the optimal cut-off value of the blood signature index. Logistic regression analysis was used to evaluate the outcomes based on the identified variables and other clinically relevant variables (odds ratio [OR], 95% confidence interval [CI]). After univariate analysis, selected variables with a *P* value < 0.10 were considered for inclusion in multivariate regression analysis to investigate the factors related to SAT and MVI. In the multivariate regression model, the *P* value was set at 0.05. In addition, in the multivariable logistic model, the Hosmer-Lemeshow goodness of fit test was also applied. The nomogram was drawn using the results of the multivariable logistic regression model for SAT and MVI. The predictive accuracy of the models was measured using the C-index, quantifying the level of agreement between the predicted probabilities and the actual possibility of having the event of interest, and the bootstrap estimate of slope shrinkage [46]. The Bootstrap resampling method was chosen for the internal validation of the predictive models’ selecting 1000 repetitions. DCA was performed to determine the clinical application value of the nomogram models by calculating the net benefits at each risk threshold probability [47].

### Categorization of patients with different conventional staging Systems

Eligible patients were categorized according to four conventional staging systems (the BCLC staging system, the 8^th^ edition of the AJCC TNM classification system (HCC and ICC) and the CNLC staging system). We conducted a group-stratified analysis to compare the discriminative ability of the nomogram with that those of the other staging systems in the training and the validation sets and were evaluated by the C-index. The larger the C-index was, the more accurate was the prognostic prediction.

### Statistical analysis

The Mann-Whitney U test was used to compare continuous variables between two patient groups. The chi-squared test and two-tailed Fisher's exact test were used for the comparison of categorical variables data between two groups. Continuous variables are expressed as medians and ranges, and categorical variables data are expressed as numbers and percentages. The OS and RFS were calculated with the Kaplan-Meier and Log-rank method using GraphPad Prism 8.0 software. R version 3.6.1 (http://www.r-project.org/) was used for ROC curve analysis, LASSO logistic regression, nomogram generation, C-index assessment, calibration plot generation, DCA, and clinical impact curve analysis. The rest of the analyses were conducted using SPSS statistical software version 24.0 (IBM Corporation, Armonk, NY). In all analyses, *P* < 0.05 was considered to indicate statistical significance.

### Synopsis

There is little information on the surgical outcomes of and prognostic factors for cHCC from previous research. Using the clinical data obtained at West China Hospital, the authors discovered prognostic factors of this malignancy for cHCC. Moreover, a nomogram was established by combining clinical risk factors using least absolute shrinkage and selection operator (LASSO) regression that can objectively and accurately predict the preoperative risks of individualized satellite nodules and microvascular invasion.

## Supplementary Material

Supplementary Figures

Supplementary Table 1
