# Supplementary material for: Integrated nomograms to predict overall survival and recurrence-free survival in patients with combined hepatocellular cholangiocarcinoma (cHCC) after liver resection
Source: Aging (Albany NY). 2020 Aug 13;12(15):15334–58. doi: 10.18632/aging.103577 (PMC7467372; doi:10.18632/aging.103577)
Supplement: Supplementary Figures [file aging-12-103577-s002..pdf]

SUPPLEMENTARY FIGURES

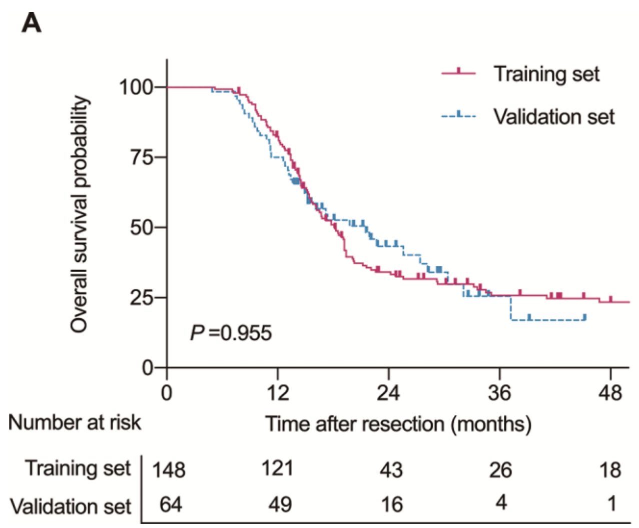

Supplementary Figure 1. (**A**). Kaplan-Meier analysis of overall survival (OS) for combined hepatocellular cholangiocarcinoma (cHCC) in the training and validation sets. (**B**). Kaplan-Meier analysis of recurrence-free survival (RFS) for cHCC in the training and validation sets. The number at risk refers to the number of patients who have not relapsed at the corresponding time point.

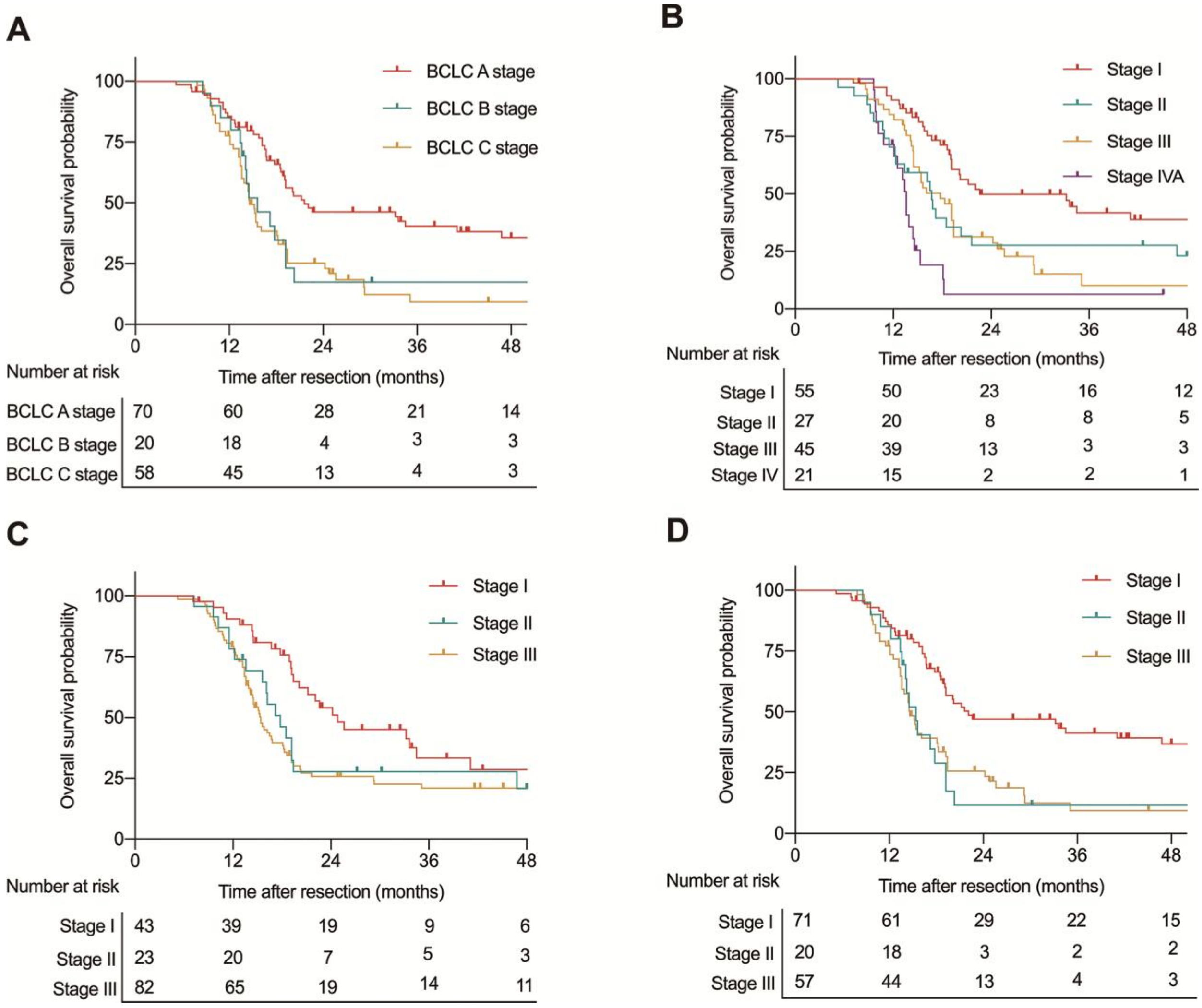

Supplementary Figure 2. Kaplan-Meier analysis of overall survival (OS) categorized by the BCLC staging systems (**A**), American Joint Committee on Cancer (AJCC) HCC TNM staging systems (**B**), American Joint Committee on Cancer (AJCC) ICC TNM staging systems (**C**), and China liver cancer (CNLC) staging systems (**D**).

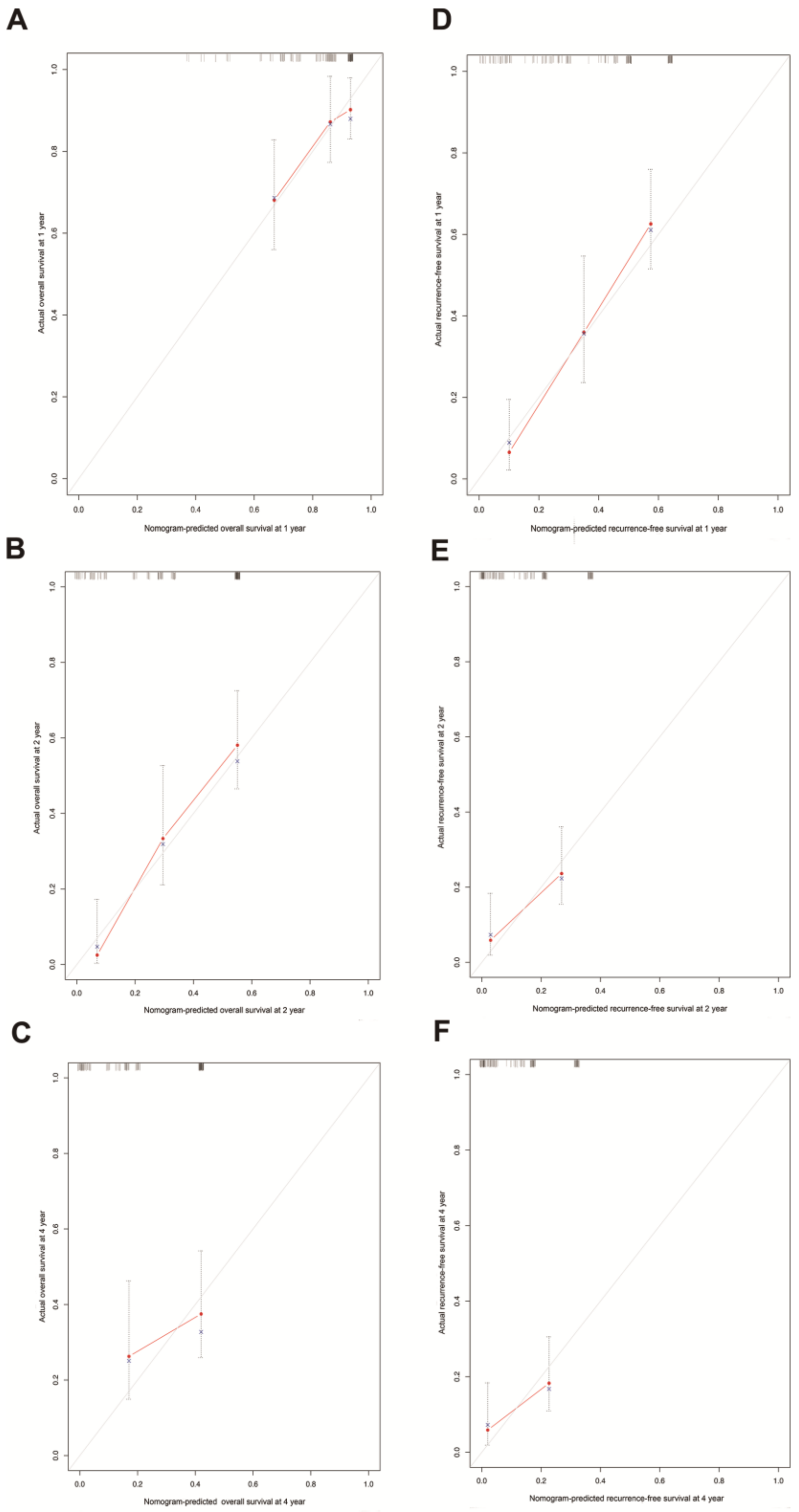

Supplementary Figure 3. The calibration curve for predicting patient 1-year, 2-year and 4-year overall survival (OS) in the training set (**A**, **B**, **C**) and 1-year, 2-year and 4-year recurrence-free survival (RFS) in the training set (**D**, **E**, **F**). The nomogram-predicted probability of overall survival is plotted on the x-axis; actual overall survival is plotted on the y-axis.

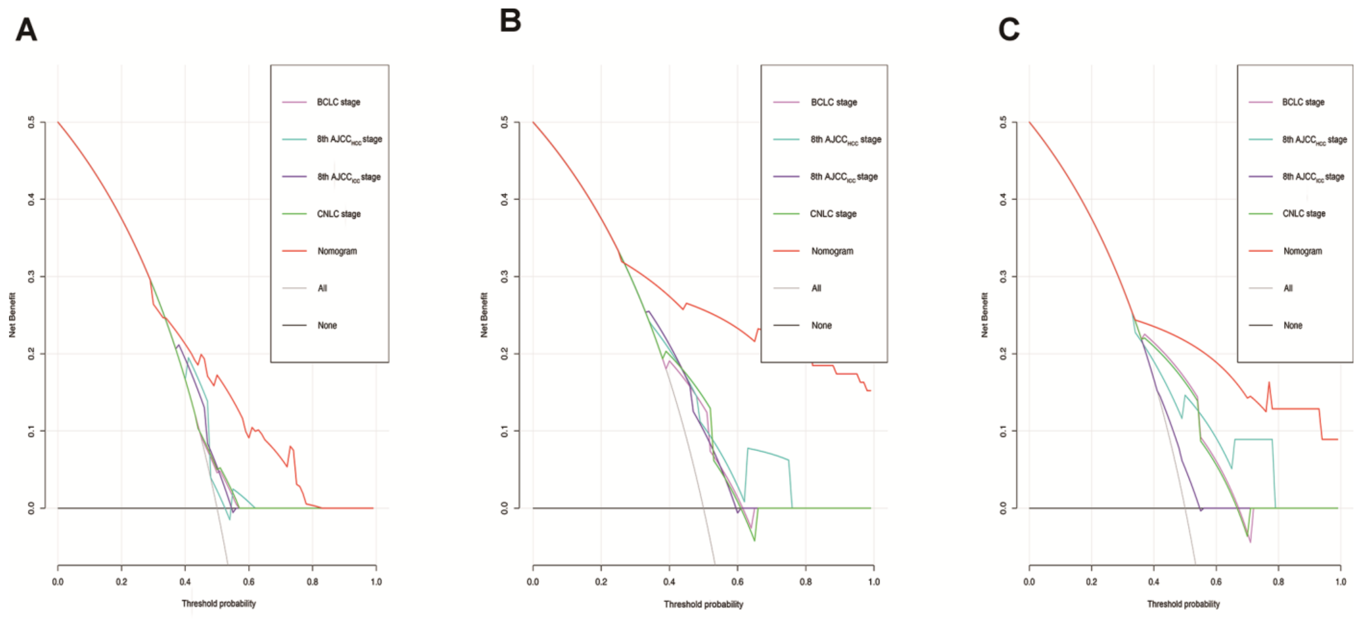

Supplementary Figure 4. Decision curve analysis (DCA) was used to compare the clinical net benifit to cHCC patients of our nomogram compared with the BCLC staging, 8th AJCC HCC staging systems, 8th AJCC ICC staging systems, and China liver cancer (CNLC) staging systems in terms of survival at 1-year (A), 2-years (B), 4-years (C).

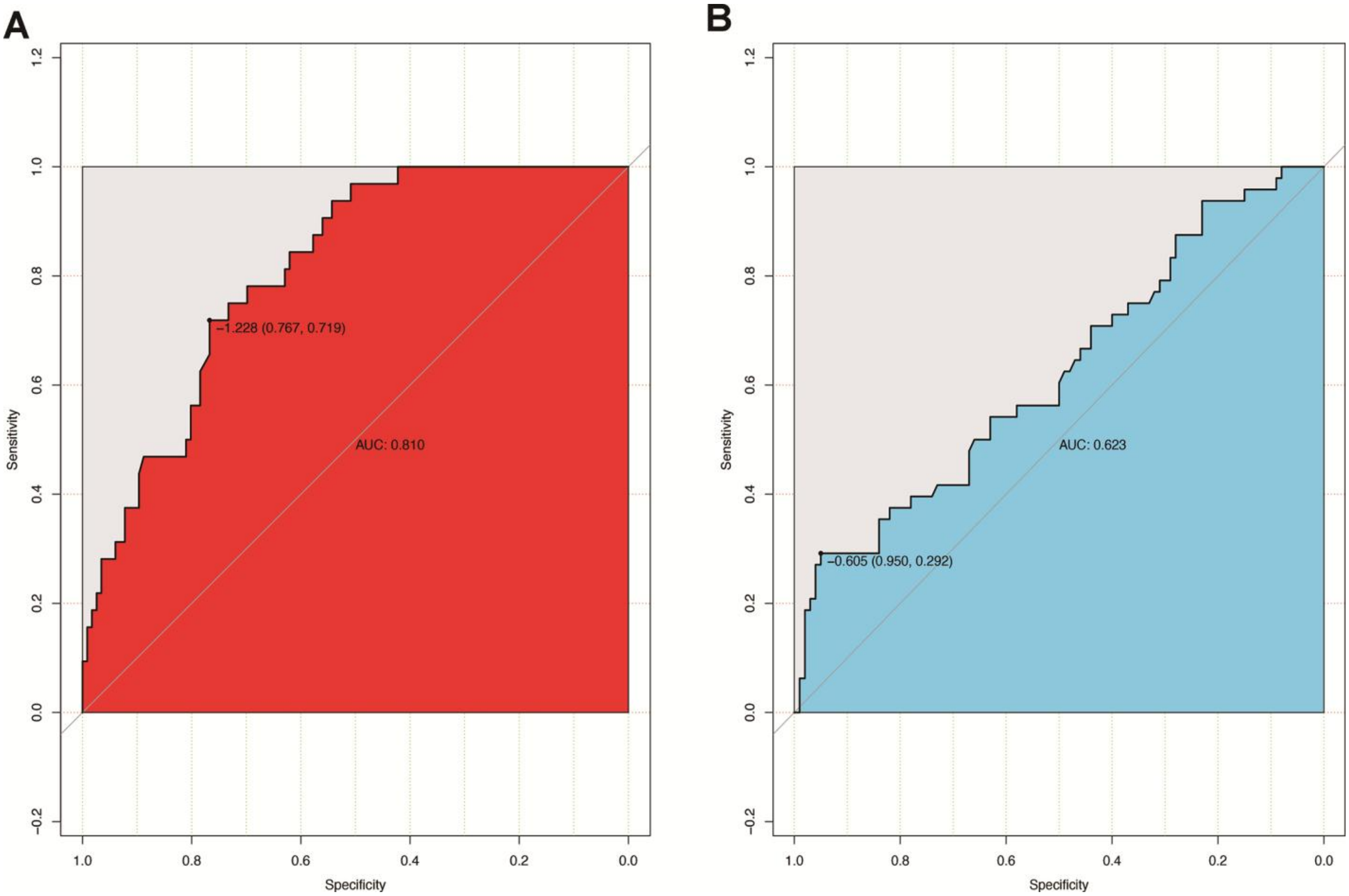

Supplementary Figure 5. Receiver operating characteristic (ROC) curve analysis of the blood signature for SAT (**A**, **red**) and MVI (**B**, **blue**). The areas under the curve (AUCs) were 0.810 and 0.623, respectively. The optimal cut-off score was -1.228 for SAT and -0.605 for MVI.
