# Supplementary material for: Integrated nomograms to predict overall survival and recurrence-free survival in patients with combined hepatocellular cholangiocarcinoma (cHCC) after liver resection
Source: Aging (Albany NY). 2020 Aug 13;12(15):15334–58. doi: 10.18632/aging.103577 (PMC7467372; doi:10.18632/aging.103577)
Supplement: Supplementary Table 1 [file aging-12-103577-s001..pdf]

SUPPLEMENTARY TABLE

Supplementary Table 1. Coefficients for each blood index in the LASSO regression models on SAT and MVI status.

Satellite nodules presence		MVI presence	
**Index**	**coefficient**	**Index**	**coefficient**
APTT	7.26E-02	PT	3.72E-02
PLT	-6.55E-04	TBIL	3.21E-02
INR	5.23E-01	ALB	-1.63E-02
TBIL	-2.57E-02		
AST	-2.61E-03		
GLB	3.06 E-02		
ALB	-8.12E-02		
CA19-9	8.64E-04		
HBV-DNA	1.053		

Abbreviations: SAT, satellite nodules; MVI, microvascular invasion; LASSO, Least Absolute Shrinkage and Selection Operator; APTT, activated partial thromboplastin time; PLT, platelet; INR, international normalized ratio; TBIL, Total bilirubin; AST, aspartate transaminase; GLB, Globulin; ALB, albumin; CA19-9, carbohydrate antigen 19-9; PT, Prothrombin Time.
